# The addition of fluoxetine to cognitive behavioural therapy for youth depression (YoDA-C): study protocol for a randomised control trial

**DOI:** 10.1186/1745-6215-15-425

**Published:** 2014-11-04

**Authors:** Christopher G Davey, Andrew M Chanen, Sue M Cotton, Sarah E Hetrick, Melissa J Kerr, Michael Berk, Olivia M Dean, Kally Yuen, Mark Phelan, Aswin Ratheesh, Miriam R Schäfer, G Paul Amminger, Alexandra G Parker, Danijela Piskulic, Susy Harrigan, Andrew J Mackinnon, Ben J Harrison, Patrick D McGorry

**Affiliations:** Orygen, The National Centre of Excellence in Youth Mental Health, 35 Poplar Road, Parkville, VIC 3052 Australia; Centre for Youth Mental Health, The University of Melbourne, 35 Poplar Road, Parkville, VIC 3052 Australia; Melbourne Neuropsychiatry Centre, Department of Psychiatry, The University of Melbourne, Level 3, Alan Gilbert Building, 161 Barry St, Carlton South, VIC 3053 Australia; Department of Psychiatry, The University of Melbourne, Level 1 North, Main Block, Royal Melbourne Hospital, VIC 3050 Kragujevac, Australia; IMPACT Strategic Research Centre, School of Medicine, Deakin University, PO Box 281, Geelong, VIC 3220 Australia; Florey Institute of Neuroscience and Mental Health, 30 Royal Parade, Parkville, VIC 3052 Australia; Australian Mathematical Sciences Institute, The University of Melbourne, Building 161, Parkville, VIC 3052 Australia

**Keywords:** Adolescence, Antidepressants, Cognitive behavioural therapy, Depression, Fluoxetine, Selective serotonin reuptake inhibitors, Youth

## Abstract

**Background:**

The aim of the Youth Depression Alleviation–Combined Treatment (YoDA-C) study is to determine whether antidepressant medication should be started as a first-line treatment for youth depression delivered concurrently with psychotherapy. Doubts about the use of medication have been raised by meta-analyses in which the efficacy and safety of antidepressants in young people have been questioned, and subsequent treatment guidelines for youth depression have provided only qualified support.

**Methods/Design:**

YoDA-C is a double-blind, randomised controlled trial funded by the Australian government’s National Health and Medical Research Council. Participants between the ages of 15 and 25 years with moderate to severe major depressive disorder will be randomised to receive either (1) cognitive behavioural therapy (CBT) and fluoxetine or (2) CBT and placebo. The treatment duration will be 12 weeks, and follow-up will be conducted at 26 weeks. The primary outcome measure is change in the Montgomery-Åsberg Depression Rating Scale (MADRS) after 12 weeks of treatment. The MADRS will be administered at baseline and at weeks 4, 8, 12 and 26. Secondary outcome measures will address additional clinical outcomes, functioning, quality of life and safety.

**Trial registration:**

Australian and New Zealand Clinical Trials Registry ID: ACTRN12612001281886 (registered on 11 December 2012)

## Background

Mental illnesses are the ‘chronic diseases of the young’ [1], and the mental illness that causes most disability in young people is major depressive disorder (MDD) [2]. The peak period for the incidence of depression (or onset of new cases) is from 15 to 29 years of age [3], and about 70% of people will have a subsequent episode of depression after their first episode [4]. Consequently, depression affects a larger proportion of the total life course than any other chronic condition [5]. It causes more impairment (measured in disability-adjusted life-years) than any other illness in high- and middle-income countries, and it is projected to be the major cause of disability internationally in all income groups by 2030 as the burden of infectious disease declines [6]. Youth depression is associated with disruption in development that has effects across the life course, including underachievement in education, underemployment, welfare dependency and diminished number and quality of friendships and intimate relationships [7–9]. The rapid developmental increase in the incidence of depression in adolescence and young adulthood, together with evidence that depression is a neuroprogressive disorder and that effective treatments can be neuroprotective [10], means that establishing effective treatments for this age group is of the utmost importance.

### The effectiveness of antidepressant medications

The effectiveness of antidepressant medications for child and adolescent depression was brought into question by a meta-analysis by Whittington and colleagues [11]. They included both published and unpublished randomised control trials in their meta-analysis and reported that only fluoxetine demonstrated a favourable risk–benefit profile and that sertraline, paroxetine, citalopram and venlafaxine had risk–benefit profiles which were either weak or unfavourable. In a subsequent meta-analysis, Bridge and colleagues [12] reached similar conclusions. They reported a mean effect size of 0.25 (95% CI, 0.16 to 0.34) for antidepressant medications compared with placebo in the treatment of child and adolescent depression, with the fluoxetine trials showing an almost twofold greater effect size of 0.46 (95% CI, 0.29 to 0.62) [12]. In a recent Cochrane review [13], which included trials of all selective serotonin reuptake inhibitors (SSRIs) and newer classes of antidepressants, the authors confirmed that the effects of antidepressant medications for youth depression were small but significant, with rates of remission increasing from 380 per 1,000 for placebo to 448 per 1,000 for medication. It is unclear why fluoxetine appears to be the most effective of the SSRIs in this population, given their similar pharmacodynamics. It might be that fluoxetine’s considerably longer half-life tempers the effects of inconsistent adherence to medication, which can be particularly problematic for young people [14]. Other evidence suggests that the difference is likely to be artefactual. In their recent Cochrane review [13], the authors implemented subgroup analysis to robustly investigate whether the effects of medication were modified by the type of antidepressant. They found no evidence for this hypothesis and concluded that it was premature to assert that fluoxetine is the most effective antidepressant for adolescent depression—especially given that there have been no head-to-head trials.

Concerns about the effectiveness of antidepressants for youth depression have led to a focus on the quality of the trials completed to date. Many of the trials in which investigators have examined antidepressant medication in young people have been industry-sponsored. They have been efficacy trials designed with high degrees of internal validity and have been conducted in highly selected patient populations enrolled in university research centres. They have not been designed to test the effectiveness of the medications in ‘real-world’ clinical populations [15]. Generalising the results from industry-sponsored efficacy trials is problematic and has led to a call for simple clinical trials in real-world patients that will aid practical clinical decision-making [16]. In addition, few studies have investigated treatment effectiveness in young people with depression up to the age of 25. The period from puberty to 25 years of age is continuous in a neurodevelopmental sense, with studies showing that important brain maturational processes start in puberty and continue up to the age of about 25 [17]. As the peak period for the onset of first episodes of depression [3], the period is a key time for effective interventions. Studies that include both children and adolescents—as many of the antidepressant trials do—are likely to confound different developmental pathologies. Childhood-onset depression, which is often a concomitant of an adverse family environment [18], is not a strong predictor of recurrent depression in adulthood [19]. Adolescent-onset depression, in contrast, is continuous with depression that has its onset in young adulthood, with the first depressive episodes in both groups often heralding lifelong recurrent illness [4].

### The safety of antidepressant medications

Additional concerns have been raised about the safety of antidepressants in young people. In 2004, the US Food and Drug Administration (FDA) published the results of a meta-analysis of placebo-controlled trials of antidepressant medications in more than 4,400 children and adolescents. They concluded that the medications doubled the risk of suicidal ideation and behaviour (4% versus 2%) [20]. There were no actual deaths in any of the studies. Subsequent meta-analyses have broadly supported these findings. Bridge and colleagues [12] reported an absolute rate of suicidal behaviour of 3% in those prescribed antidepressant medication, compared with 2% in those who received placebo. They found, however, that the risk difference of 1% was not significant (95% CI, −0.1% to 2%). The authors of the recent Cochrane review found that the rate of suicidal behaviour did increase significantly, from 25 per 1,000 for adolescents treated with placebo to 40 per 1,000 for adolescents treated with antidepressant medication [13]. In response to these increased risks in adolescents, the FDA mandated ‘black box’ warnings for all antidepressants [21], which was followed by similar warnings issued by the Medicines and Healthcare Products Regulatory Agency in the United Kingdom [22], the European Medicines Agency [23] and the Therapeutic Goods Administration in Australia [24]. In 2006, the FDA expanded their warning to include young people up to the age of 25 on the basis of an extended examination of placebo-controlled trials that included almost 100,000 participants in total [20], further emphasising the importance of studies that include patients in this age range.

It is not clear why young people treated with antidepressant medications may be more likely to develop increased suicidality, with hypotheses including that SSRIs induce mixed symptoms in depressed youth with latent bipolarity [25] or that these medicines induce akathisia in a subset of patients [26]. There is some evidence that the small increase in risk can be managed within a supportive clinical framework. The Treatment for Adolescents with Depression Study (TADS), a large trial funded by the US National Institute of Mental Health that included fluoxetine and cognitive behavioural therapy (CBT) (and which is discussed further below), showed that the rate of suicidal behaviour in the group treated with fluoxetine and CBT was half the rate of the group treated with fluoxetine alone during the 12-week trial period [27]. Furthermore, there has been some suggestion that the reported increase in suicidality might not apply to all antidepressants, with the authors of a recent meta-analysis finding no increase in suicidal thoughts or behaviours in young people treated with fluoxetine [28]. The Cochrane review, however, not only confirmed the increase in suicidal thoughts and behaviours associated with antidepressant treatment but also revealed that the type of antidepressant did not modify this risk [13].

Concerns about the efficacy and safety of antidepressant medications for youth depression have led to the development of treatment guidelines that provide only qualified support for their use. The United Kingdom’s National Institute for Clinical Excellence guideline states that psychotherapies should be offered as first-line treatment and that medication should be considered only for moderate to severe depression when there has been insufficient response to psychological therapy [29]. In Australia, the National Health and Medical Research Council guideline also states that psychotherapies should be the first-line treatment, but that fluoxetine should be considered when the patient’s depression is severe, when there has been no response to psychotherapy or when psychotherapy is unavailable [30]. The Australian guideline acknowledges the uncertainty about the use of medications for young people, stating, ‘Many clinically relevant questions remain unanswered by current research evidence, including when a health professional should recommend medication, for whom and at what severity of depression, for how long it should be taken, and how best to monitor progress’ (p 53).

### The effectiveness of psychotherapy and combined treatments

Psychotherapy has proved to be an effective therapy for depression; it is as effective as medication in the short-term treatment of adult patients [27]. Over 80% of published trials of therapy for adolescent depression have studied CBT [31], a manualised, time-limited treatment aimed at improving depressive symptomatology by targeting depressogenic thoughts and related behaviours. Early evidence suggested that CBT was among the most effective treatments for adolescent depression; it showed an effect size approximately twice that of medication [32]. Recent evidence suggests, however, that the effectiveness of psychotherapy for adolescent depression might also be more modest than previously thought, especially in clinical patient groups with severe illness [33].

Evidence derived from studies that have included medication and CBT arms has provided inconsistent support for the effectiveness of one treatment over another or for combined treatment, and it suggests a need for further studies. In TADS [16], the researchers compared four treatments for adolescents with depression: (1) fluoxetine alone, (2) CBT alone (without pill-placebo treatment), (3) combination of fluoxetine and CBT and (4) pill placebo with medical management. The participants were aged from 12 to 17 years and had a mean Clinical Global Impression Scale (CGI) severity score of 4.8, corresponding to ‘markedly ill’ [34]. After 12 weeks of treatment, patients receiving fluoxetine (in the medication-only and combined fluoxetine–CBT treatment arms) showed significantly greater response compared with patients in the CBT and placebo groups, whereas the CBT-alone group did not differ from the placebo group on any measure. The absence of placebo-pill treatment in the group treated with CBT alone, and thus the lack of patient and clinician blinding, was a significant limitation of the study [35], especially given that young people with depression respond particularly robustly to placebo-pill treatment [36]. The benefits of combined treatment versus therapy alone have been examined in other studies, which have produced inconsistent findings. In a study by Melvin and colleagues [37], adolescent participants were allocated to receive sertraline, CBT (also without placebo) or their combination. The study, which was unblinded and had low power to detect differences between treatment groups, did not show any advantage for combined treatment, though CBT alone was more effective than sertraline alone. Mandoki and colleagues [38] observed no difference in depression improvement between participants treated with CBT and venlafaxine compared with CBT and placebo. In a number of small studies, researchers have compared combined treatment with therapy alone for comorbid depression and substance use disorders and have largely found no difference between these treatment approaches [39–42], although Riggs and colleagues [40] observed an improvement in the group treated with CBT and fluoxetine on one depression measure, but not on another (and no difference in substance use outcomes). The authors of a recent Cochrane review [43] found no evidence that combination therapy was more effective than psychological therapy alone for adolescent depression based on clinician-rated remission (OR, 1.82; 95% CI, 0.38 to 8.68).

Overall, the evidence suggests that both CBT and antidepressants are modestly effective for youth depression. The modest effectiveness of both treatments suggest advantages for combined treatment, but this approach currently has limited support. Despite the practical importance of the clinical question, there is still genuine clinical equipoise as to whether first-line management of youth depression should be CBT alone or combined treatment. Although depression is the single greatest cause of morbidity during adolescence and young adulthood, the optimal treatment for the illness in this age group remains a source of considerable uncertainty. With the present protocol, we seek to address this issue by implementing a simple, practical clinical trial in a ‘real-world’ clinical population.

## Methods/Design

### Study design and ethical approval

The study design is a 12-week, parallel-group, double-blind, randomised control trial in which participants with moderate to severe MDD will be allocated to treatment with either CBT and fluoxetine (CBT + FLX) or CBT and placebo (CBT + PBO). The primary hypothesis is that, after 12 weeks of treatment (the trial endpoint), the CBT + FLX group will show greater improvement than the CBT + PBO group in depressive symptoms compared to baseline. The primary outcome measure we will use is the Montgomery-Åsberg Depression Rating Scale (MADRS), an observer-rated depression scale used widely in depression treatment trials because it is both efficient to administer and psychometrically sound [44]. Our secondary hypotheses are that the CBT + FLX group will show greater improvement than the CBT + PBO group on secondary clinical outcomes, functioning, quality of life (QoL) and safety measures at 12 weeks. Assessments will be completed at baseline and at weeks 4, 8 and 12, with follow-up assessment completed by telephone at week 26. All participants will receive manualised CBT, which will be offered weekly to participants for a period of 12 weeks. The study was approved by the Melbourne Health Human Research Ethics Committee (EC00243) in October 2012.

### Study setting

The study is being conducted in the Youth Mood Clinic (YMC), which is part of the Orygen Youth Health Clinical Program (OYHCP), and in associated *headspace* centres. OYHCP is a youth public mental health service for 15- to 25-year-olds who live in western metropolitan Melbourne, Australia. YMC treats about 150 patients each year, about 80% of whom have a diagnosis of MDD. The clinic is staffed by psychiatrists, psychiatry trainees and therapists, who are mainly clinical psychologists, but also include social workers and occupational therapists. Patients are reviewed regularly by their treating doctors, and therapists deliver weekly CBT and case management. All patients have access to after-hours crisis care and inpatient treatment when needed. About half of the YMC patients are being treated for depression for the first time, and comorbid diagnoses are common: 30% have comorbid anxiety disorders, and 12% have substance use disorders. The median length of treatment at the clinic is 6 months.

The *headspace* centres provide enhanced primary care services funded by the Australian government and are located throughout the country. Local centres in the northern and western regions of Melbourne are managed by the OYHCP. The *headspace* centres are staffed by psychiatrists, general practitioners and allied health professionals who manage patients between 12 and 25 years of age with high-prevalence mental illnesses, mainly depression and anxiety. The model of care is similar to that at YMC, with patients being reviewed regularly by medical practitioners and attending weekly CBT sessions with their therapists.

### Inclusion and exclusion criteria

The criteria for inclusion in and exclusion from the study are designed to reflect the real-world characteristics of the young people with depression who present to the clinical centres. The inclusion criteria are (1) age between 15 and 25 years at the time of commencement of the intervention; (2) diagnosis of MDD based on the Structured Clinical Interview for DSM-IV Axis I Disorders, Patient Edition (SCID-I/P) [45]; (3) score of 20 or higher on the MADRS, indicating depression of at least moderate severity; and (4) ability to provide written informed consent (including both adequate intellectual capacity and fluency in the English language).

The exclusion criteria are (1) lifetime or current SCID-I/P diagnosis of a psychotic disorder, (2) lifetime or current SCID-I/P diagnosis of bipolar I or II disorder, (3) acute or unstable medical disorder that would interfere with treatment, (4) current pregnancy, (5) severe disturbance such that the young person would be unable to comply with the requirements of informed consent or comply with the study protocol, (6) current treatment with an antidepressant medication taken for at least 2 weeks and (7) previous treatment with fluoxetine that was either ineffective or poorly tolerated.

### Enrolment and randomisation

After participants provide their written informed consent (which will also be obtained from a parent or legal guardian if the participant is younger than 18 years of age), they will undergo a baseline assessment in which they will be screened for eligibility. Participants will be randomised to either the CBT + FLX or CBT + PBO group in a 1:1 ratio, sequentially as they become eligible for randomisation. The randomisation will be stratified by site (OYHCP and *headspace* centres), age (≤18 and >18 years) and sex, and randomly permuted blocks (of sizes 2, 4 and 6) will be programmed into the electronic case report form by an independent statistician. The investigators, research assistants, study biostatistician, clinicians and participants will all be blinded with respect to randomisation. Only the pharmacist will be aware of the group to which the participant has been allocated. Participants can be unblinded in emergency situations when it is important that medical staff know which medication the participant has received. If unblinded, the participant’s treatment in the study will then be discontinued. Participants whose study medication treatment is discontinued will continue to be assessed at the scheduled times, provided they have not withdrawn their consent. Participants will be unblinded by the treating doctor after the 12-week period so that the doctor can make appropriate clinical decisions about the participant’s continuing management.

### Study medication

Participants will be commenced on one 20-mg tablet of fluoxetine or one tablet of the placebo pill. The medication can be increased to fluoxetine 40 mg daily (or to two placebo pills) if there is a poor clinical response at any time after the first 4 weeks. The medication will be prescribed by the treating doctor after the baseline visit is completed and the participant has been randomised. The trial medications will be supplied every 4 weeks following medical review, with a total of three dispensations per participant. Adherence to medication will be assessed by self-report and a pill count.

Participants will not be allowed to be treated with antipsychotic medications or mood-stabilising medications during the 12 weeks of the trial. They can be treated with sedative hypnotics (benzodiazepines and benzodiazepine-like medications) as clinically indicated. The use of medications for treatment of medical illnesses is permitted. During the follow-up period (between 12 and 26 weeks), which will be uncontrolled, treatments can be prescribed as clinically indicated. The administration of all medications (including investigational products) will be recorded during the 12-week trial and at the 26-week follow-up examination.

### Cognitive behavioural therapy

CBT is the most studied psychotherapy for young people with depression, with more than 30 trials in child and adolescent patients [33] and over 100 trials in adult patients [46]. Treatment manuals used in these trials have targeted either paediatric or adult populations, however, and are not especially suitable for young people ranging in age from late adolescence to young adulthood. To bridge this gap, we have developed a CBT manual for youth depression in collaboration with clinicians from YMC and the *headspace* centres. Recent evidence supports the use of ‘modular’ CBT manuals [47] encompassing a flexible and formulation-driven approach to therapy rather than the more linear approach prescribed by older treatment manuals. The manual that has been developed consists of 14 modules comprising 7 core modules and 7 targeted modules. The core modules are delivered to all participants and represent the central components of CBT. They cover psychoeducation, monitoring emotions, behavioural activation, chain analysis, identifying negative thoughts, restructuring negative thoughts and relapse prevention. The skills developed in each of the core modules can be revised and practiced over the course of several sessions. The targeted modules are used as suggested by the participant’s difficulties, such as if the participant has particular problems with insomnia or social anxiety. Thus, not every targeted module is relevant for every participant.

The therapist will record details of each of the sessions that the participant attends, including documenting the modules delivered. Weekly 50-minute sessions will be offered for 12 weeks. Participants will be expected to attend each week, but not all of the participants are likely to be able to attend every session. When possible, the therapist will attempt to reschedule sessions that the participant is unable to attend. All therapists will attend regular supervision sessions with senior clinicians. The fidelity of CBT treatment will be assessed in terms of adherence and competence. Each CBT session will be recorded, and the deidentified recording will be stored on a secure server. Random sessions from the course of therapy for each participant will be independently rated using CBT fidelity scales.

### Outcome measures

In addition to the primary outcome measure (the MADRS), we will use a number of measures to assess secondary clinical outcomes, functioning, QoL and safety.

#### Secondary clinical measures

The secondary clinical outcome measures we will use are the Quick Inventory of Depression Symptomatology–Self Report [48], the CGI Scale severity score [34], the CGI Scale improvement score [34] and the Patient Global Impression improvement score [49].

#### Functioning and quality-of-life measures

The functioning and QoL outcome measures we will use are the Social and Occupational Functioning Scale [50], the Social Adjustment Scale–Self-Report [51] and the Quality of Life Enjoyment and Satisfaction Questionnaire–Short Form [52].

#### Safety measures

Safety will be assessed using the Suicidal Ideation Questionnaire [53] and the Columbia Suicide Severity Rating Scale [54].

### Predictors, moderators and mediators of treatment response

We will explore factors that might explain individual differences in treatment outcomes. These factors will include (1) clinical variables, such as the duration of the depressive episode, the number of episodes and family history; (2) treatment variables, such as the quality of therapy and the number of therapy sessions attended; and (3) measures of personality, bipolarity and substance use. In addition, participants will have the option to provide blood samples and to undergo baseline brain imaging. For the blood sampling, 30 ml of blood will be drawn from consenting participants at baseline and at week 12, and the blood will be processed and stored for later analyses. We are particularly interested in examining inflammatory biomarkers and understanding how these might relate to treatment outcomes [10]. For imaging analysis, consenting participants will undergo a 1-hour magnetic resonance imaging session, during which they will complete a number of tasks. These tasks will include a cognitive reappraisal paradigm, an emotional face-matching task and a self-referential task. The data will be analysed to determine whether brain imaging variables could predict treatment outcomes, including whether they moderate or mediate outcomes.

### Assessment of suicidal ideation and behaviours

Monitoring participants closely for the presence of suicidal thoughts and behaviours is an important component of the study protocol. Participants will be clinically assessed for the presence of suicidal ideation and behaviours at each meeting with their doctor (which will occur at least every 4 weeks) and at their weekly therapy sessions. The assessment will be carried out using a structured tool, the Columbia Suicide Severity Rating Scale [54]. In addition, participants will complete the Suicidal Ideation Questionnaire [53] at the research assessment sessions. If the young person indicates the presence of suicidal thinking in a research assessment, the research assistant will liaise with the participant’s treating doctor or therapist, and the clinician will ensure that an appropriate management plan is in place. If participation in the study interferes with appropriate clinical management of risk of harm to the individual (as adjudged by the treating clinicians), then that person’s participation in the study will be discontinued.

### Statistical methods and determination of sample size

Primary analyses will be undertaken on an intention-to-treat basis, including all participants as randomised, regardless of treatment actually received. The CBT + FLX group will be compared with the CBT + PBO group using a planned contrast of change from baseline to the week 12 endpoint on the basis of MADRS scores within a mixed-model repeated measures analysis [55]. Stratification variables will be evaluated and retained in analyses where they are measured as significant or quasi-significant. An unconstrained variance–covariance matrix will model within-individual dependencies. Transformation of scores, including categorisation, may be undertaken to meet distributional assumptions and accommodate outliers. Comparisons of changes in MADRS scores from baseline to other time points will be undertaken as secondary analyses.

Models for binary outcomes analogous to the primary analysis approach will be used to compare the remission rates and other dichotomous outcomes between the two treatment arms at endpoints and other occasions of measurement. Relative and absolute risk reduction, number needed to treat [56] and other relevant indices will be calculated for these outcomes. In analyses of scaled secondary variables, methods comparable to those employed in the primary analysis will be used. In exploratory analyses, the effects of moderators and mediators of treatment will be examined. Safety data will be compared between treatment arms—in particular, the rates of suicide-related adverse events—using Fisher’s exact test. All tests will be conducted using a two-sided α level of 0.05 and 95% confidence intervals.

The primary outcome measure is change on the MADRS at 12 weeks. On the basis of large, previously published fluoxetine trials of adolescent depression, we have estimated the pre–post effect size for fluoxetine to be 0.35. This value is less than the mean effect size of 0.5 reported in previous trials, as we have taken into account the modest effectiveness of the CBT administered in both arms. To sufficiently power the study, and assuming a correlation of 0.5 between baseline and 12-week endpoint measurements, a total sample size of 260 participants will be required. For the secondary outcome measure, remission at 12 weeks, there will be 74% power to detect an improvement in the rate of remission of at least 15% in the CBT + FLX arm, assuming remission in the CBT + PBO arm of 20% (consistent with remission in the TADS study).

## Discussion

Depression is the single greatest cause of morbidity among adolescents and young adults and has a significant influence on the development of an affected person’s social relationships and vocational skills. Its effects are felt across the life course, with substantial costs to the affected individuals, their families and the community. The optimal treatment for depression during adolescence and early adulthood is a source of considerable uncertainty, creating a dilemma for clinicians as to what they should recommend to their patients as first-line treatment. In the present study, we seek to address this issue by conducting a simple, practical clinical trial, the outcomes of which have the potential to substantially influence clinical practice. If the trial shows that fluoxetine provides additional clinical benefits when administered in combination with CBT, it will support first-line use of combined treatment for young people with moderate to severe depression. If, on the other hand, no evidence of any additional benefit is demonstrated, then the current advice to withhold medication will be given empirical support.

## Trial status

The study commenced enrolling participants in February 2013, and enrolment was continuing at the time of manuscript submission.

## Abbreviations

CBT: Cognitive behavioural therapy
CGI: Clinical Global Impression Scale
DSM-IV: *Diagnostic and Statistical Manual of Mental Disorders, Fourth Edition*
FDA: US Food and Drug Administration
FLX: fluoxetine
MADRS: Montgomery-Åsberg Depression Rating Scale
MDD: Major depressive disorder
OYHCP: Orygen Youth Health Clinical Program
PBO: placebo
SCID-I/P: Structured Clinical Interview for DSM-IV Axis I Disorders, Patient Edition
SSRI: Selective serotonin reuptake inhibitor
TADS: Treatment for Adolescents with Depression Study
YMC: Youth Mood Clinic
YoDA-C: Youth Depression Alleviation–Combined Treatment study.
